# Effects of truck transportation and slaughtering on the occurrence of prednisolone and its metabolites in cow urine, liver, and adrenal glands

**DOI:** 10.1186/s12917-019-2069-4

**Published:** 2019-09-18

**Authors:** Pierluigi Capra, Marta Leporati, Carlo Nebbia, Stefano Gatto, Alberto Attucci, Gandolfo Barbarino, Marco Vincenti

**Affiliations:** 1 Istituto Zooprofilattico Sperimentale del Piemonte, Liguria e Valle d’Aosta, Torino, Italy; 2 Centro Regionale Antidoping e di Tossicologia “A. Bertinaria”, Orbassano (Torino), Italy; 3 Dipartimento di Scienze Veterinarie, Università degli Studi di Torino, Grugliasco (Torino), Italy; 4 Azienda Sanitaria Locale di Collegno e Pinerolo - ASL TO3, Collegno (Torino), Italy; 5 Azienda Sanitaria Locale di Cuneo, Mondovì – ASL CN1, Cuneo, Italy; 6 Regione Piemonte – Direzione Sanità Pubblica, Torino, Italy; 7 Dipartimento di Chimica, Università degli Studi di Torino, Via Pietro Giuria, 7, 10125 Torino, Italy

**Keywords:** Glucocorticoids, Prednisolone, Stress, Slaughtering, Endogenous biosynthesis, Urine, Liver, Adrenal glands, 20β-dehydroprednisolone

## Abstract

**Background:**

The recognition of illegal administration of synthetic corticosteroids in animal husbandry has been recently challenged by the case of prednisolone, whose occasional presence in the urine of bovines under strong stressful conditions was attributed to endogenous biosynthesis, not to exogenous administration. The study of the natural stress sources possibly inducing endogenous prednisolone production represents a stimulating investigation subject. The biochemical effects of transportation and slaughtering were verified in untreated cows by studying the possible occurrence of prednisolone and its metabolites in urine, liver and adrenal glands, and the cortisol/cortisone quantification.

**Results:**

Cortisol, cortisone, prednisolone and its metabolites were measured in urine, collected at farm under natural micturition and then at the slaughterhouse. The study was performed on 15 untreated cows reared in different farms at the end of their productive cycle. 2–3 days after the first urine collection, the animals were transported by trucks to the abattoir, slaughtered, and subjected to a second urine sampling from the bladder. Specimens of liver and adrenal gland were also collected and analysed by means of a liquid chromatography-tandem mass spectrometry (LC-MS/MS) validated method. The stressful conditions of transportation and slaughtering proved to increase considerably the urinary levels of cortisol and cortisone as compared to those collected at farm. Prednisolone was detected in the urine collected at the slaughterhouse of two cows only, at a concentration level (≈0.6 μg L^− 1^) largely below the official cut off (5.0 μg L^− 1^) established to avoid false non-compliances. These two animals exhibited the highest urinary cortisol levels of the series. Prednisolone and prednisone were also detected in the adrenal glands of a different cow. Prednisolone metabolites were not detected in any urine, liver, and adrenal gland sample.

**Conclusion:**

Within the constraints of the condition adopted, this study confirms the sporadic presence of prednisolone traces (2 samples out of 15) and the consistently increased concentration of cortisone and cortisol in the urines collected from cows subjected to truck transportation and subsequent slaughtering. No prednisolone metabolites were detected in any liver and adrenal gland samples, nor in urine specimens, unlike what was previously reported for cows artificially stressed by pharmacological treatment.

## Background

**Fig. 1:**
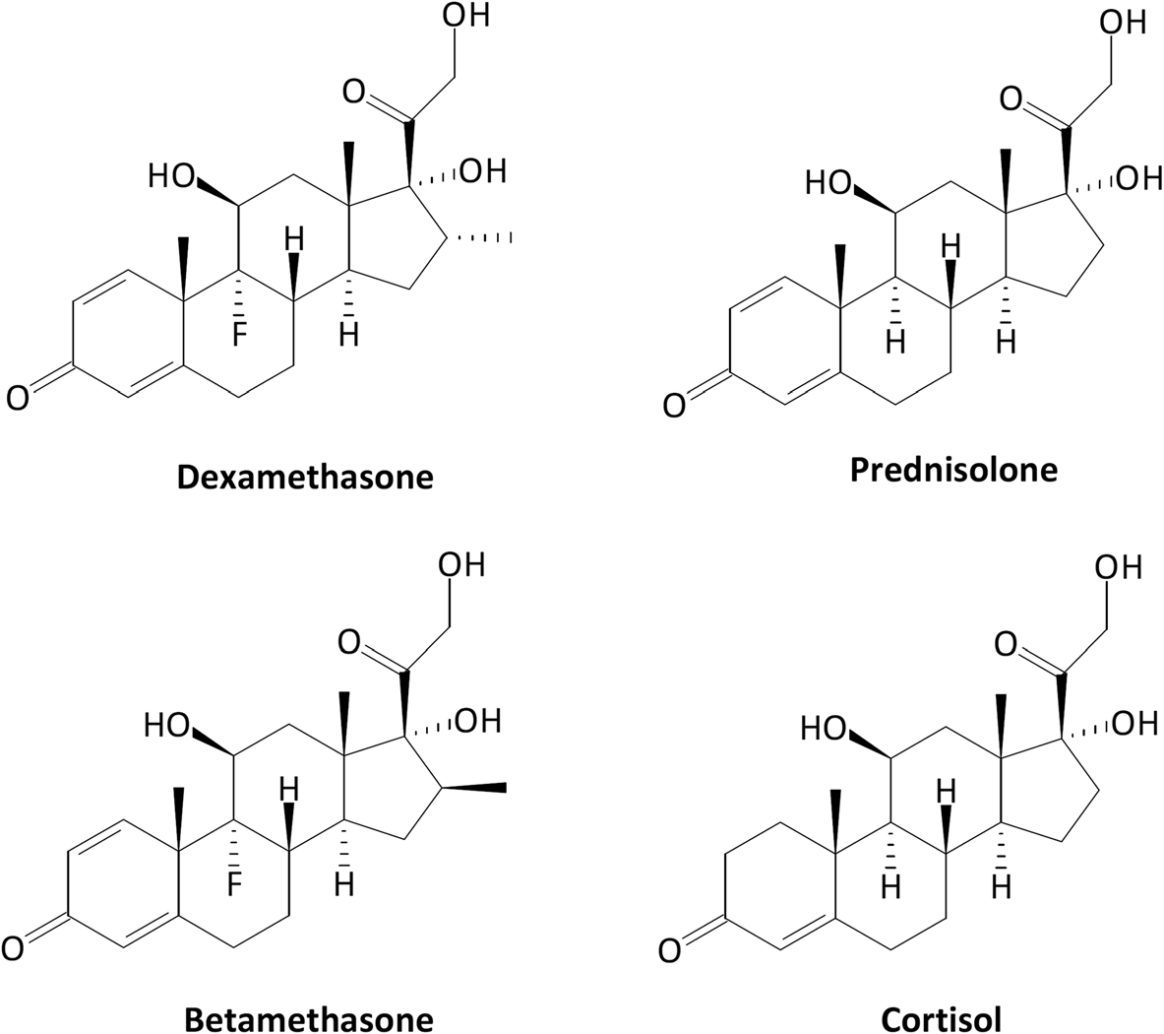
Molecular structures of the most commonly used synthetic corticosteroids, dexamethasone, betamethasone, and prednisolone, highlighting the similarity between the latter and cortisol

A wide range of steroidal molecules have been synthesized to replicate (with enhancement in efficiency and duration) the anti-inflammatory properties of corticosteroids. Among the synthetic “corticosteroid-like” molecules, prednisolone is the one with the chemical structure most similar to the natural adrenal steroids. Unlike betamethasone, dexamethasone and several other synthetic molecules, prednisolone does not contain any halogen atom and differs from cortisol uniquely for the presence of a second double bound on the A ring (Fig. 1).

Prednisolone and the other synthetic corticosteroids can be used in veterinary therapy under strict medical control to treat musculoskeletal and skin diseases, allergic reactions and shock, and other pathologies [1, 2]. These drugs are also misused to conceal animal illness or as growth-promoters, in which case they are administered at sub-therapeutic doses. Their illegal use is monitored in the EU area through National Residue Control Plans, established according to the 96/23 EC directive.

In the last years, several research groups have independently obtained experimental evidence consistent with the possibility of endogenous/natural biosynthesis of prednisolone, both in vitro [3] and in live animals [4, 5]. Based on these evidences, the Italian Ministry of Health [6] issued a technical note establishing a precautionary threshold concentration of 5 μg L^− 1^ for prednisolone in bovine urine. This threshold was subsequently used as a legal cut-off to assess its unequivocal exogenous origin. An identical threshold was proposed later by the researchers of the E.U. Reference Laboratory [3].

In the frame of the project “Investigation on prednisolone and its metabolites in meat and milk producing bovines, assessment of endogenous biotransformation through “in vivo” and “in vitro” experiments” supported by “Regione Piemonte–Direzione Sanità - Prevenzione Veterinaria”, a study on 131 non treated cows was first carried out throughout the years 2011/2012 in order to evaluate the possible natural presence of prednisolone in their urine specimen. The presence of prednisolone at trace concentration levels (i.e., 0.1–0.3 μg L^− 1^) was found in 7 animals only [7]. It is worth noting that all “positive” animals were reared in loose housing systems, thereby experiencing significant stress from the restraining and sample collection procedures, as was reflected also in an increased urinary cortisol concentration with respect to the animals usually kept tethered. In the frame of the project mentioned above, a characterization of the urinary excretion profile of prednisolone was also performed in both healthy finishing bulls and cows after the intramuscular (i.m.) administration of a therapeutic schedule [8]. Among the results of this kinetic study, it should be mentioned that very low urinary cortisol and cortisone levels were recorded at any checked time-point, along with a consistent occurrence of 20β-dihydroprednisolone and, more sporadically, other reduced or oxidized metabolites of prednisolone and prednisone.

Endogenous prednisolone was found in the urines and adrenal glands [9] of dairy cows subjected to the distinctive state of stress consequent to their transport and slaughter. More recently [10], the plasma pharmacokinetic and urinary excretion profiles of relevant glucocorticoids were investigated in cattle subjected to either exogenous prednisolone treatment or the administration of synthetic ACTH analogue tetracosactide hexaacetate to induce a stress allegedly leading to endogenous prednisolone formation. The results of this study point to the determination of the urinary prednisolone/cortisol ratio as an expedient strategy to discriminate between endogenous and exogenous prednisolone.

The present work was aimed at the evaluation of the possible occurrence of prednisolone in urine, together with the levels of cortisol and cortisone, arising from the severe stressful conditions of transport and slaughtering operation. A second objective was to assess the possible presence of prednisolone metabolites (20α-dihydroprednisolone, 20β-dihydroprednisolone, 6β-hydroxyprednisolone, 20β-dihydroprednisone) which have been so far associated with either therapeutic or illicit administration of the drug. The last unprecedented goal was to target the same prednisolone precursors and metabolites in liver and adrenal glands as possible organs of prednisolone biosynthesis.

## Results

### Adrenal gland method validation

#### Specificity

The SRM chromatographic profiles obtained from 10 adrenal glands samples did not reveal the presence of any significant signal (S/*N* < 3) at the relative retention time typical of the studied compounds and the internal standards, with the exception of cortisol and cortisone.

#### Linearity

The calibration curves obtained for all the analytes showed good fit and linearity over the entire range of interest (Table 1), with the exception of cortisol and cortisone. For these analytes the dynamic linear range proved narrower than that initially planned (0.5–2.5 μg L^− 1^ and 0.5–5.0 μg L^− 1^, respectively). The most likely explanation for the calibration curve deflection is an incomplete ionization that may occur at the highest concentrations. In practice, the real samples with cortisol or cortisone concentration exceeding the linear range had to be properly diluted in order to fall back into it.

**Table 1:** Validation results of the analytical method developed for adrenal glands. Intraday precision and accuracy were calculated at three concentration levels (low, intermediate, high)

Analyte	Instrumental linearity range (μg L^− 1^)	R^2^	Intraday precision (CV%)				Accuracy (bias %)			
			0.5 μg Kg^− 1^	2.0 μg Kg^− 1^	10.0 μg Kg^− 1^	20.0 μg Kg^− 1^	0.5 μg Kg^− 1^	2.0 μg Kg^− 1^	10.0 μg Kg^− 1^	20.0 μg Kg^− 1^
6β-hydroxyprednisolone	0.10–1.0	0.9953	–	6	8	8	–	+ 4	0	−7
20α-dihydroprednisolone	0.10–1.0	0.9906	–	9	5	12	–	− 13	− 5	+ 5
20β-dihydroprednisolone	0.10–1.0	0.9919	–	11	10	12	–	+ 4	+ 1	−1
20β-dihydroprednisone	0.10–1.0	0.9900	–	8	9	7	–	−9	−6	+ 5
Prednisolone	0.025–0.50	0.9988	6	8	7	–	+ 10	+ 6	+ 4	–
Prednisone	0.025–0.50	0.9995	11	5	9	–	+ 7	+ 3	+ 1	–
Cortisol	0.50–2.5	0.9962								
Cortisone	0.50–5.0	0.9983								

#### Precision and trueness

Intraday data on precision and accuracy are reported in Table 1. The results show satisfactory intra-day repeatability, as the percent variation coefficient (CV%) was lower than 15% for all the spiked analytes at low, intermediate, and high concentration levels. Intraday results also exhibited optimal trueness, as the percent bias fell within few percent units in almost all cases, with maximum experimental errors of − 13 and + 10%.

### Real samples results

All the data collected for real samples are reported in Table 2. In urine samples collected at farm, cortisol and cortisone concentrations ranged from non-detectable to 3.5 μg L^− 1^ with the only exception of animal 13 whose sample contained 24 μg L^− 1^ of cortisone and 52 μg L^− 1^ of cortisol. Particularly laborious sampling operations were recorded at farm for this animal that might explain the unusually high levels for cortisol and cortisone detected in this specific sample. None of the specimens collected at farm contained measurable amounts of prednisone or prednisolone. Truck transportation and slaughtering indeed induced a sharp increase in both urinary cortisol (range 1.1÷145 μg L^− 1^, median 21 μg L^− 1^) and cortisone (range from non-detectable to 53 μg L^− 1^, median 12 μg L^− 1^). In two samples (animals 2 and 15), that displayed the highest concentrations of both cortisol and cortisone, the presence of prednisolone was also detected at concentrations of 0.57 and 0.60 μg L^− 1^, respectively.

**Table 2:** Analytical results obtained for urine and adrenal gland samples. Unavailable samples are indicated with N.A., while N.D. stays for “not detectable”

Urine samples								
Animal	Cortisone (μg L^−1^)		Cortisol (μg L^− 1^)		Prednisone (μg L^− 1^)		Prednisolone (μg L^− 1^)	
	Farm	Slaughterhouse	Farm	Slaughterhouse	Farm	Slaughterhouse	Farm	Slaughterhouse
1	N.D.	16	N.D.	23	N.D.	N.D.	N.D.	N.D.
2	1.5	49	N.D.	98	N.D.	N.D.	N.D.	0.57
3	N.A.	6.5	N.A.	9.1	N.A.	N.D.	N.A.	N.D.
4	N.A.	18	N.A.	45	N.A.	N.D.	N.A.	N.D.
5	N.D.	N.D.	0.68	1.1	N.D.	N.D.	N.D.	N.D.
6	N.D.	5.3	1.5	12	N.D.	N.D.	N.D.	N.D.
7	1.1	1.6	2.1	2.6	N.D.	N.D.	N.D.	N.D.
8	N.D.	3.9	N.D.	7.8	N.D.	N.D.	N.D.	N.D.
9	N.D.	8.5	N.D.	15	N.D.	N.D.	N.D.	N.D.
10	0.69	25	1.1	44	N.D.	N.D.	N.D.	N.D.
11	N.D.	16	N.D.	21	N.D.	N.D.	N.D.	N.D.
12	1.4	3.6	3.5	5.2	N.D.	N.D.	N.D.	N.D.
13	24	12	52	31	N.D.	N.D.	N.D.	N.D.
14	0.60	16	1.0	32	N.D.	N.D.	N.D.	N.D.
15	1.6	53	2.4	145	N.D.	N.D.	N.D.	0.60
Adrenal glands samples								
Animal	Cortisone (μg Kg^−1^)		Cortisol (μg Kg^−1^)		Prednisone (μg Kg^−1^)		Prednisolone (μg Kg^−1^)	
1	476		150		N.D.		N.D.	
2	682		905		N.D.		N.D.	
3	1102		738		N.D.		N.D.	
4	615		230		N.D.		N.D.	
10	485		145		N.D.		N.D.	
11	286		46		N.D.		N.D.	
12	437		185		3.4		4.2	
13	43		12		N.D.		N.D.	
14	38		7.5		N.D.		N.D.	
15	85		23		N.D.		N.D.	

One liver sample (animal 11) was found to contain measurable amounts of cortisol (0.88 μg Kg^− 1^). The other specimens were negative to all the measured analytes.

**Fig. 2:**
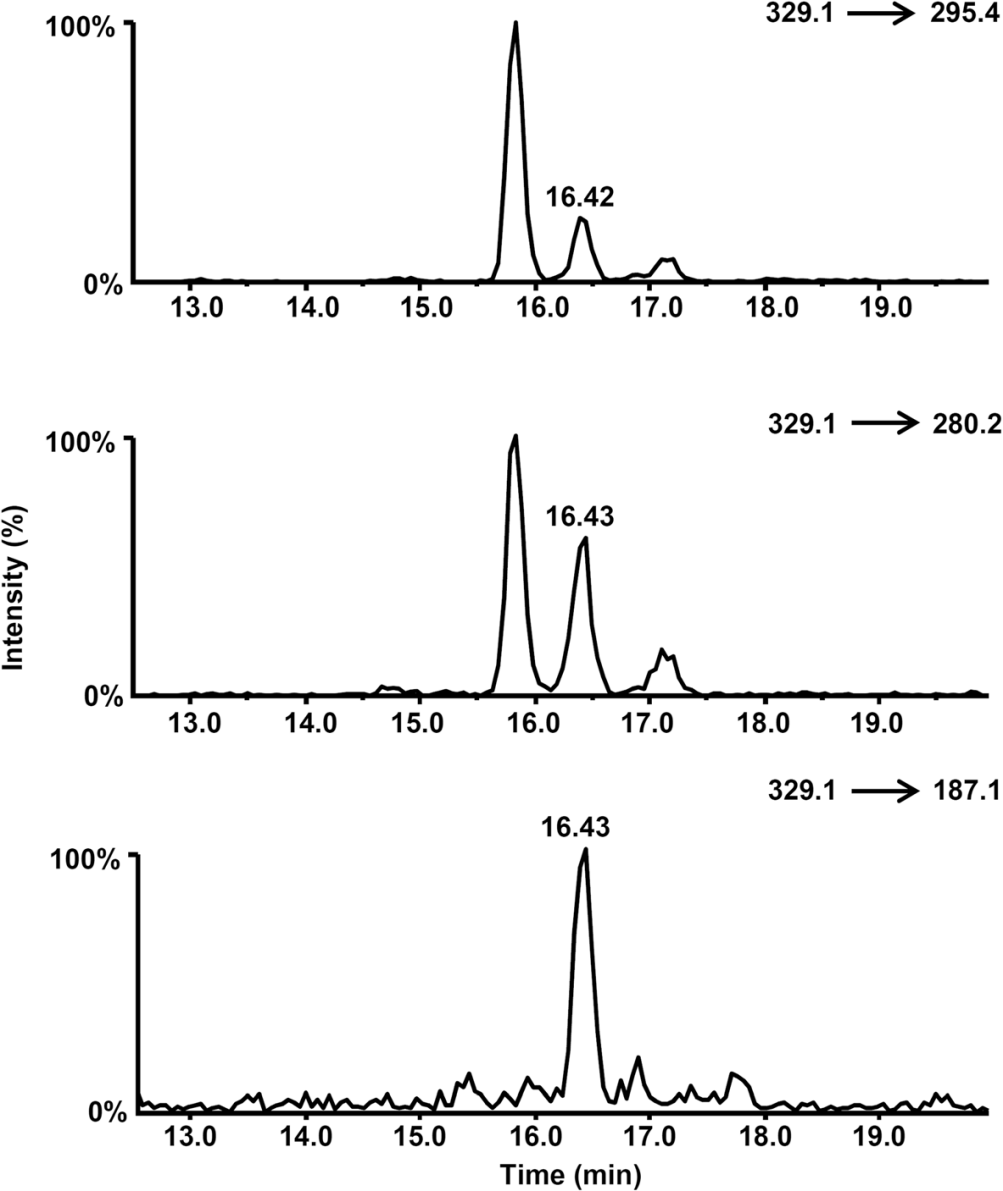
Selected ion chromatograms of the SRM transitions between the [M-H-(HCHO)]^−^ precursor ion of prednisolone and the target (m/z 295) and qualifier fragment ions (m/z 280, 187). The chromatographic retention time of prednisolone is 16.43 min

Adrenal specimens exhibited uneven levels of cortisone and cortisol; in one animal (#12), prednisone and prednisolone (Fig. 2) were detected at 3.4 μg Kg^− 1^ and 4.2 μg Kg^− 1^ respectively), but the corresponding urines proved negative for both compounds.

## Discussion

The data from this study further confirm that, under conventional breeding conditions, no prednisolone or very limited amounts of it can be synthesized in cows and excreted in urine. Interestingly, no traces of prednisolone/prednisone nor their examined metabolites were found in the urines collected from cows sampled at farm and also negligible levels of cortisol and cortisone were recorded. In agreement with the results of previous studies [4, 9, 11], our findings confirm that stressful events such as truck transportation followed by slaughtering increase the urinary levels of cortisol and cortisone up to several times as compared to those collected at farm. In our study, such a dramatic increase of cortisone and cortisol levels was associated with the production of prednisolone in only two animals out of fifteen, notably those whose urine contained the highest concentrations of cortisol (145 and 98 μg L^− 1^). Remarkably, the prednisolone concentration measured in these two cases (0.57 and 0.60 μg L^− 1^) was about one order of magnitude below the official threshold of 5.0 μg L^− 1^ set by the Italian Ministry of Health. In both “positive” urine samples, the cortisol/prednisolone ratio appeared to be reversed with respect to that displayed by animals submitted to exogenous prednisolone administration for either therapeutic or growth-promoting purposes, when the presence of prednisolone and its metabolites was combined with low cortisol levels [8].

An important result of the present study is that neither the urine samples collected at farm nor those taken from slaughtered animals were found to contain measurable amounts of 20β-dihydroprednisolone, namely the most abundant urinary metabolite of prednisolone. Our results are corroborated by a previous investigation [11] in which neither 20α- nor 20β-dihydroprednisolone were detected in urines collected in “naturally stressed” bovines, namely 64 cows and 34 bulls after slaughtering. Likewise, Leporati et al. [12] reported that no 20β-dihydroprednisolone nor any other prednisolone metabolites were found, even at trace levels, in the urine samples from 108 allegedly stressed cows involved in the “Bataille des Reines”, a traditional bloodless tournament in which animals are allowed to fight spontaneously to assess dominance. In contrast, the cited study by De Clercq and coworkers [11] reported the detection of prednisolone, along with its 20α- and 20β-dihydroderivatives, in the urine of “artificially stressed” cows, subjected to i.m. treatment with tetracosactide, a synthetic ACTH analogue. Although the urine of the cows undergoing such a pharmacological treatment exhibited the occurrence of natural corticoids levels similar to those found in slaughtered cows, no plausible explanation was provided to justify the apparent discrepancy between absence and presence of prednisolone in naturally and artificially stressed animals, respectively. More recently, van Meulebroek et al. [10] reported the detection of 20β-dihydroprednisolone in all urine specimens collected from cows subjected to prednisolone treatment, under either therapeutic or (oral) growth-promoting schedule, as well as in cows treated (by i.m. injection) with tetracosactide to induce a pharmacological stress. In the latter paper, however, the relative increase in urinary cortisol and cortisone concentrations with respect to T0 values (i.e., prior to tetracosactide administration) was uniquely reported, so that a direct comparison with the absolute levels of natural corticoids recorded in naturally stressed cows [11] is not feasible. Based on the evidences presently available, the reasons for the seemingly different urinary pattern profile of prednisolone metabolites between naturally and artificially stressed cows remain to be elucidated. As a matter of fact, besides the cases of pharmacologically-induced stress, the presence of 20β-dihydroprednisolone and other prednisolone metabolites in the cattle urine has been so far consistently associated only with the exogenous administration of the parent drug, either following a growth-promoting protocol or a therapeutic schedule [8, 10, 13].

Earlier investigations disclosed that in-vitro cortisol incubation with bacteria [11] and bovine hepatic S9 preparations [3] resulted in the progressive decrease of cortisol along with the generation of prednisolone. This finding suggests that the liver is possibly the involved organ for the endogenous prednisolone synthesis. In the present study, neither prednisolone nor its dihydro-derivatives could be detected in any liver sample of the slaughtered cows, including the two of them displaying the presence of urinary prednisolone. In addition, traces of cortisol corresponding to about 1 μg Kg^− 1^ were detected in only one liver sample. This important evidence apparently indicate that transport and slaughtering stressing conditions are not associated with any significant increase of hepatic cortisol concentration, partly contradicting what was observed in the in-vitro investigations using much higher cortisol concentrations (5 mM).

In a study characterized by an experimental design similar to the present one [9], measurable amounts of prednisolone were detected in about one third of the analysed adrenal glands along with high levels of cortisol. In our study, only one sample was found to contain prednisolone (3.4 μg Kg^− 1^) along with relatively high concentrations of cortisol (185 μg Kg^− 1^) and cortisone (437 μg Kg^− 1^), but further five samples with similar or even much higher concentration of both natural corticoids did not show the presence of prednisolone at any level. In agreement with the observations of Bertocchi et al. [9], no correlation was found between the presence of prednisolone in the adrenal glands and in urine, suggesting that the endogenous synthesis of prednisolone is a complex phenomenon possibly involving different organs and tissues other than liver and adrenal glands.

## Conclusions

This study confirms the sporadic presence of prednisolone traces (2 out 15) and the consistently increased concentration of cortisone and cortisol in the urines collected from cows subjected to truck transportation and subsequent slaughtering. The detected prednisolone levels were found much below the official cut off of 5.0 μg L^− 1^ established to avoid false non-compliances. Based on the results of this and other studies [11, 14], it may also be concluded that, in cows subjected to various natural sources of stress, the occasional positive finding of urinary endogenous prednisolone is never matched by the presence of 20β-dihydroprednisolone or any other prednisolone metabolite. It is also evident that this irregular endogenous prednisolone biosynthesis cannot be directly attributed to a specific organ (liver or adrenal glands) and further research is needed to ascertain the possible sites of endogenous prednisolone synthesis in naturally stressed cows.

An evident limitation of the present study is the lack of standardization for the cattle transportation conditions to the abattoir, that introduces a potentially uncontrolled influencing factor possibly enhancing the correlated stress variability. While this limitation was inherently associated with the study commitment to investigate only cows at the end of their (re) productive cycle, making slaughtering by no means compelled by the scientific purpose to obtain more homogeneous experimental conditions, it is apparent that future development of the present study should more accurately model the stressing factors associated to cattle transport, including truck type, duration, distance, road conditions, and temperature and humidity environment.

## Methods

### Urine, liver and adrenal gland collection

Fifteen clinically healthy Piedmontese or Friesian cows (age range 8–14 years, average weight around 600 kg) were enrolled in the study, which was conducted between mid- April and May. The monthly temperature in Piedmont, averaged for location, day and hour was 9.5 °C in April and 12.8 °C in May [15]. All cows were at the end of their (re) productive cycle and ready for slaughter. They were bred in two conventional farms (40–200 animals, respectively) located in the Turin and Cuneo provinces of Piedmont (northern Italy) and kept in tie stall barns. Farms were selected according to personal records and breeder formal declaration that the cows had not been subjected to any drug treatment in the last 30 days prior to sampling procedures, at least. Urine samples were first collected at farms under conditions of natural micturition, taking care to prevent faecal contamination. After 2 to 3 days, the animals were transported [16] to the abattoir (40 and 80 km journey from the two farms to the abattoir, on plain roads using single level trucks, with no facilities for measuring temperature and humidity). Transport was carried out according to Council Regulation (EC) N. 1/2005 and cows were slaughtered 30 to 45 min after the arrival. The animals were first desensitized with a captive-bolt pistol and then exsanguinated in the conventional way. Post-mortem sampling of liver and adrenal glands was performed by licensed veterinarians within 15 min from slaughtering, together with urine from the urinary bladder. After collection, all samples were immediately refrigerated (put on ice), frozen at − 20 °C within 4 h, and subsequently conferred to the analytical laboratory. The whole study was conducted under the strict control of the Regional Veterinary Public Services of Torino and Cuneo (Piedmont, Italy).

### Chemicals, reagents, and standard solutions

Diethylether and acetonitrile together with cortisone, cortisol, prednisone, and prednisolone analytical standards were supplied by Sigma–Aldrich (St. Louis, MO, USA). All solvents were of analytical grade. 20α-dihydroprednisolone, 20β-dihydroprednisolone, 6β-hydroxyprednisolone, and 20β-dihydroprednisone were supplied by Steraloids (Newport, RI, USA). Cortisol D2, cortisone D2, and prednisolone D6 were purchased from C/D/N Isotopes Inc. (Pointe-Claire, Quebec, Canada). Triamcinolone acetonide D6 was from RIVM (Bilthoven, The Nederlands). Sodium hydroxide and hydrochloric acid were supplied by Carlo Erba Reagenti (Milan, Italy). Betaglucuronidase/aryl-sulfatase was from Roche Diagnostics (Mannheim, Germany). Ultrapure water was obtained by a Milli-Q Millipore system (Bedford, MA, U.S.A.).

Stock standard solutions of the analytes were prepared in acetonitrile at a concentration of 1 mg L^− 1^ and stored at − 20 °C in the dark. Working acetonitrile solutions containing all the analytes at different concentrations were prepared by proper dilution. The working solutions were used to spike negative urine samples for the validation of the analytical method.

### Sample preparation

#### Urine

Sample preparation was conducted as reported elsewhere (Vincenti et al. 2012). Briefly, 5 mL urine samples were subjected to a liquid/liquid extraction at pH = 8.5–9.5 with diethylether, after β-glucuronidase/arylsulfatase deconjugation. After centrifugation, the supernatant organic phase was transferred into a 10 mL glass tube and evaporated to dryness under nitrogen with 40 °C heating. The residue was dissolved in 50 μL of a water:acetonitrile mixture (70:30 v/v) solution and transferred into the analytical vials for the analysis.

#### Liver/adrenal gland

Sample aliquots of 2.5 g were homogenized in 5 ml acetate buffer and then added with 50 μL of the internal standard mix solution at 0.1 ng L^− 1^. Further acetate buffer 0.1 M was added (5 mL; pH = 5) and the extraction was carried out by 5 min shaking plus 5 min ultrasonic bath. After centrifugation (3500 rpm for 5 min), the aqueous phase was extracted with 10 mL of *tert*-butyl methyl ether. The organic phase was transferred into a 10 mL glass tube and evaporated to dryness under a gentle stream of nitrogen and mild heating (50 °C). For adrenal glands, a further washing step was executed to remove fat. The residue, dissolved in 2 mL acetonitrile, was washed with 2 mL of hexane and dried again. The residue was dissolved again in 50 μL of a water:acetonitrile mixture (70:30 v/v) solution and transferred into the analytical vial.

#### Instrumental analysis

All the samples were analysed by LC-MS/MS using the method previously described by Cannizzo et al. [17], and further implemented by Vincenti et al. [7] and Leporati et al. [13]. An Agilent 1100 LC was interfaced to an Applied Biosystems API 4000 triple quadrupole mass spectrometer (Applied Biosystems Sciex, Ontario, Canada), operating in atmospheric pressure chemical ionization (APCI). Each sample was analysed twice and quantified by means of a calibration curve, using internal standard correction.

For the urine and adrenal gland samples that were found positive to prednisolone at concentrations lower than the CCα (i.e., matching the prednisolone reference standard in terms of retention time and presence and relative abundance of the product ions), the correct identification of prednisolone was further confirmed by reprocessing the samples under a different MS/MS acquisition method. Following the procedure described by Savu and coworkers [18], the APCI source was operated in the negative ion mode under high declustering potential (DP), which induced an “in-source” fragmentation of the deprotonated molecular ion (M-H)^−^ yielding a loss of formaldehyde (HCHO; 30 Da) from the (C_21_) hydroxymethyl group and the formation of the [M-H-(HCHO)]^−^ precursor ion with considerable abundance. The latter was subsequently fragmented under selected reaction monitoring (SRM) conditions. The experimental settings are reported in Table 3.

**Table 3:** Instrumental parameters for prednisone and prednisolone using the confirmation method based on negative APCI and “in source” collisional fragmentation of the deprotonated molecular ion to yield the [M-H-(HCHO)]^−^ precursor ion

MS parameters			CUR = 20 psi					CAD = 2 psi		
			GS1 = 50 psi					T = 300 °C		
			NC = - 3 V					ihe = ON		
SRM protocol			Target		Qualifier 1			Qualifier 2		
Analyte	Precursor Ion	DP (V)	Fragment	CE (V)	Fragment	(qualifier 1/quantifier %)	CE (V)	Fragment	(qualifier 2/quantifier %)	CE (V)
Prednisolone	329.1	−96	295.4	−25	280.2	90%	−31	187.1	81%	−25
Prednisone	327.2	−90	149.2	−39	285.4	45%	−25	270.3	26%	−28

*CUR* curtain gas, *GS1* nebulizer gas, *NC* nebulizer current, *CAD* collision gas, *T* temperature, *ihe* interface heater, *DP* declustering potential, *CE* collision energy

#### Method validation

For urine and liver samples, the method was validated accordingly to 2002/657/CE Decision (2002/657 CE Decision) for prednisone and prednisolone [17]. For other analytes a simplified validation protocol was applied [7, 13].

For the screening analysis of the adrenal glands a new and simplified validation protocol was executed. This matrix is not adopted for the official controls of the Italian National Residue Plan [19]. SRM transitions with corresponding potentials for target compounds and internal standards are reported in Table 4.

**Table 4:** Instrumental conditions used for validating the analytical method and subsequently used in the screening of adrenal gland

Analyte (correspondent IS in brackets)		t_R_ (min)	Precursor Ion	DP (V)	Target		Qualifier		
					Fragment	CE (V)	Fragment	(qualifier/quantifier %)	CE (V)
6β-hydroxyprednisolone (A)		4.78	377.1	47	341.1	11	323.2	28%	15
20α-dihydroprednisolone (A)		11.28	363.3	37	345.2	15	309.3	75%	18
20β-dihydroprednisolone (A)		12.58	363.3	37	345.2	15	309.3	24%	18
20β-dihydroprednisone (A)		12.87	361.3	55	153.0	21	313.2	54%	16
Prednisolone (A)		17.16	361.3	55	343.3	16	325.3	38%	16
Prednisone (A)		17.68	359.1	70	341.1	17	313.2	41%	19
Cortisol (B)		17.72	363.2	69	121.3	33	147.3	67%	44
Cortisone (C)		18.64	361.2	83	163.4	36	105.3	14%	64
Internal standards									
A	Prednisolone D6	16.95	367.1	48	330.3	16	–	–	–
B	Cortisol D2	17.68	365.2	65	121.8	39	–	–	–
C	Cortisone D2	18.61	363.1	67	165.1	34	–	–	–

*DP* Declustering Potential, *CE* Collision Energy

Since cortisol is synthesized from cholesterol in the *zona fasciculata* of the adrenal cortex, no blank matrix for cortisol and cortisone is available, forcing us to build the calibration curves from standard solutions.

The assessment of specificity, linearity, precision, accuracy was included in the validation procedure.

#### Specificity

Ten adrenal glands were extracted and analysed as described above. The occurrence of possible interferences from endogenous substances was tested by monitoring the SRM profiles characteristic for each investigated compound, at the retention time interval expected for their elution.

#### Linearity

The instrumental linearity was studied to estimate if the quantification range of the method is encompassed by the instrumental dynamic linear range. Five concentrations of each analyte pure standard solutions (0, 25, 50, 100, 250, 500 μg L^− 1^ for prednisone and prednisolone, 0, 100, 250, 500, 750, 1000 μg L^− 1^ for prednisolone and prednisone metabolites, and 0, 0.5, 1.25, 2.5, 5, 12.5 mg L^− 1^ for cortisol and cortisone) were injected, to set up the linearity testing curves. Each level was injected in triplicate.

#### Precision and trueness

Intraday precision (expressed as percent variation coefficient, CV%) and trueness (expressed as bias%) were evaluated at three concentration levels: 0.5, 2 and 10 μg Kg^− 1^ for prednisone and prednisolone, 2, 10 and 20 μg Kg^− 1^ for prednisone and prednisolone metabolites. Five replicates of adrenal gland samples were spiked with the standard solutions at three concentration levels. Intraday precision was considered satisfactory when CV% values were below 15%. Satisfactory trueness was achieved when the experimentally determined average concentration lied within ±15% from the expected value.

## Data Availability

Raw data for calculation of method validation, tables, and figures are available from the corresponding author upon request.

## Abbreviations

ACTH: Adrenocorticotropic hormone
APCI: Atmospheric pressure chemical ionization
CCα: Decision limit
CE: Collision energy
DP: Declustering potential
LC: Liquid chromatography
MS/MS: Tandem mass spectrometry
R2: Determination coefficient
SRM: Selected reaction monitoring
